# Some Causes of Hæmaturia

**Published:** 1904-08-20

**Authors:** 


### Hospital Clinics.

### SOME CAUSES OF HÆMATURIA.

Hæmaturia is a not infrequent symptom in medical practice, and when it occurs, as it often does, without physical facts or other conclusive evidence pointing to its cause, it is apt to give rise to much perplexity. In spite of the various classical methods presented by authority this perplexity is not always readily resolved, and it extends both to the situation and the nature of the lesion. The general distinctions presented as between hæmorrhage from the kidney on the one hand and from the bladder on the other are hardly one of them of absolute application, and most clinicians will allow that they have met with cases of hæmaturia which have not been carried to a satisfactory interpretation. Hence it may possibly be useful to quote some recent records in which hæmaturia was the prominent symptom and in which bleeding did not depend on organic disease of the kidney or bladder, at least in the ordinary sense of that phrase. And first in this connection it may be well to place hæmaturia due to the *Bilharzia hæmatobia*. The South African war and modern developments in Egypt have brought large numbers of British subjects to latitudes where the parasite abounds, and it is not surprising to find that a certain proportion of the visitors become infected. Hence it has happened that quite a number of cases of hæmaturia from this cause have been reported in Britain during the last few years. The diagnosis can hardly be missed provided the practitioner bears the possibility just alluded to in mind. All that is necessary is to examine the deposit from the urine under the microscope, when the ova of the Bilharzia are readily discovered. They are of considerable size—$\frac{1}{150}$ to $\frac{1}{200}$ inch in length—oval in shape, with a well-defined outline and a bright, translucent appearance. At one end is a short sharply-pointed spine, though occasionally this is found on the lateral aspect of the ovum. Several "puzzling" cases of hæmaturia have had all the mystery attached to them promptly dissipated by the discovery of these ova in the urinary deposit. Another class of cases in which hæmaturia often gives rise to much trouble in diagnosis is infantile scurvy. According to a recent lecture by Still,[1] hæmaturia in this disease is the rule rather than the exception. In some instances albumen in small quantities is found, but no blood, and the suggestion is that this is in kind the same condition, also that both blood and albumen are consequences not of a nephritis, but of the same abnormal vascular or blood condition, whatever it may be, that determines the occurrences of other hæmorrhages in scurvy. In reference to the absence of blood and the presence of albumen, it may be remembered that a similar state of affairs has been noted in paroxysmal hæmoglobinuria, where in mild attacks albumin, but no blood, is detected in the urine. The point of importance here, however, is that it is not merely in cases where the evidences of infantile scurvy are conspicuous and easily recognised that hæmaturia occurs. If that were so its interpretation could hardly be missed. It is, on the contrary, possible that hæmaturia may be the one ruling symptom, and that excepting some general evidences of malnutrition and peevishness, there may be little or nothing beyond it to point to the diagnosis of infantile scurvy. These are the cases in which hæmaturia assumes so puzzling a character, and its appearance in an infant should certainly cause a suspicion in the direction here indicated. Again, in blood-stained urine in children, the possibily of paroxysmal hæmoglobinuria should be remembered; and further, that in such patients this symptom carries a very strong suggestion of congenital syphilis. Its occurrence in severe forms of the acquired disease has long been known, but it is only of recent years that paroxysmal hæmoglobinuria has been recognised in the inherited form of syphilis. A more frequent and simple condition in infants is the hæmaturia that attends undue acidity with uratic deposits often signalled by much screaming during the act of micturition, and readily got rid of by a few doses of potassium citrate. To revert again for a moment to hæmoglobinuria, a most curious case was recorded last year by Ensor and Wakelin Barratt.[2] The patient was a lunatic who at occasional intervals struck himself repeatedly and with great violence on the forehead, each of these attacks being followed by hæmoglobinuria. In another case the mystery of a blood-coloured urine was promptly cleared up by the discovery that the boy had been rubbing into his scalp an ointment containing carbolic acid, for the cure of ringworm. A most remarkable family history of hæmaturia has been recorded by L. G. Guthrie[3] under the term "congenital, hereditary, and family hæmaturia." Of 12 persons affected, eight were brothers, sisters, or first cousins, two were the mothers of these children, and two were their maternal uncles. Some few other examples of this condition have been noted, but though of very great interest they are at present little more than clinical curiosities. Probably little more can be said of a case of Goodhart's[4] in which the patient—a woman and a pronounced neurotic—had all the symptoms of renal calculus including the repeated passage of large quantities of blood in the urine; though at the postmortem examination which followed a nephrectomy, the kidneys, bladder, and indeed all parts of the body were absolutely healthy. Less exceptional, though by no means common, is the association of hæmaturia with influenza. Vivian Poore[5] noted three cases of this condition in 1893, but during the more recent epidemics it does not appear to have attracted much attention. A condition which might readily have been expected to give rise to hæmaturia is movable kidney. As a matter of fact, common as more or less undue mobility of the right kidney is found to be in women, it is rarely attended with any abnormal condition of the urine. Exceptionally, however, there is hæmaturia, and in a case reported by A. J. Cabot,[6] the quantity of blood was extreme, and produced an alarming condition of collapse; ultimately nephrorrhaphy was performed, and the patient made a good recovery. Another association with hæmaturia is found in appendicitis. This is somewhat puzzling, and, in a sense, one may say unfortunate. Most physicians presented with a case of acute pain in the right abdomen of more or less obscure origin would say that the occurrence of hæmaturia concluded the diagnosis in favour of renal calculus, or at least of some similar physical condition in the right kidney. Certainly in a balanced diagnosis between renal calculus on the one hand and appendicitis on the other the appearance of hæmaturia would be a highly significant fact in favour of the former. But there are experiences which show that this decision is not an absolute one. Thus Thorburn[7] relates two examples of hæmaturia in connection with appendicitis. In each the patient was a boy, and but for the presence of blood in the urine the cases were perfectly typical and straightforward; recovery followed operation and the hæmaturia disappeared. Even yet more obscure than such cases is one recorded by Spencer,[8] in which the patient, a man of 44, died after hæmaturia persisting for a fortnight, the post-mortem examination failing to discover a lesion in any part of the urinary tract. There is less difficulty in following such a clinical record as that related by Abram,[9] who, called to a case in which hæmaturia was the sole symptom, found evidences of mitral disease, and suggested therefore the development of renal embolism. It is hardly more difficult to appreciate a case noted by Schulthess,[10] where hæmaturia appeared in a boy after eating a quantity of rhubarb. The occasional instances in which blood has appeared in the urine after the administration of urotropin are doubtless in much the same category. In conclusion may be mentioned the reddish or cherry-coloured tint of the urine in cases of hæmatoporphyrinuria. This has most commonly been observed after the administration of sulphonal, but there are some few instances in which this drug can be excluded. Calvert[10] reports the condition in a case of gastric ulcer attended with hæmatemesis, W. H. Brook[11] met with it in a patient suffering from pernicious anæmia, and in some few other diseases the pigment has been detected in small amount. In one instance[12] the condition followed the use of 30 grains of sulphonal and the case had a fatal termination. Trional and tetronal have also been known to cause hæmatoporphyrinuria. The pigment is best detected by means of the spectroscope.

1 Lancet, August 13th, 1904. 2 Brit. Med. Journ., March 28th, 1903. 3 The Lancet, May 3rd, 1902. 4 Common Neuroses, p. 117. 5 Clinical Journal, Vol. II., p. 289. 6 Lancet, April 12th, 1902. 7 Medical Chronicle, November 1901. 8 Brit. Med. Journ., April 9th, 1904. 9 Lancet, August 6th, 1904. 10 Brit. Med. Journ., December 22nd, 1900. 11 Ibid. 12 Journal of Mental Science, April 1898.

